# Marketing violations involving products that compete with breastfeeding in the vicinity of early childhood education centers and health centers: a cross-sectional study based on an audit of the retail food environment, Maceió, 2022–2023

**DOI:** 10.1590/S2237-96222025v34e20240666.en

**Published:** 2025-09-29

**Authors:** Emanuelle Cristina Lins Bastos, Nicole Almeida Conde Vidal, Luan dos Santos Aragão, Gabriel Marx Assunção Costa, Jonas Augusto Cardoso da Silveira

**Affiliations:** 1 Universidade Federal de Alagoas, Programa de Pós-Graduação em Nutrição, Maceió, AL, Brazil; 2 Universidade Federal do Paraná, Departamento de Nutrição, Curitiba, PR, Brazil

**Keywords:** Food Social Space, Breast Feeding, Breast-Milk Substitutes, International Code of Marketing of Breastmilk Substitutes, Observational Study, Espacio Social y Comida, Lactancia Materna, Sustitutos de la Leche Humana, Código Internacional de Comercialización de Sucedáneos de la Leche Materna, Estudio Observacional

## Abstract

**Objective:**

Evaluate violations of the Brazilian Code of Marketing of Infant and Toddler Food, Teats, Pacifiers, and Baby Bottles (NBCAL) in the retail food environment around health centers and early childhood education centers in Maceió.

**Methods:**

This cross-sectional study, conducted between April 2022 and March 2023, audited all food retail outlets selling products regulated by the Code in the vicinity of health centers and early childhood education centers. The results were expressed as frequencies.

**Results:**

Of the 1,176 food retail outlets audited, 8.8% showed violations. The violations were concentrated in pharmacies (65.0%) and supermarkets (26.2%). When examining these two types of establishments (n=601), it was observed that 41.6% of those affiliated with retail chains exhibited violations. The probability of violation in retail chains was 6.8 times higher (95% confidence interval: 4.5-10.2) than in independent establishments. The most frequent violations occurred in follow-up formulas for early childhood children (27.5%), infant and follow-up formulas for infants (22.0%), and dairy compound products (19.7%). The promotional strategy most associated with the violations was special display (74.7%).

**Conclusion:**

The retail food environment in Maceió revealed violations of the Code, with pharmacy chains and supermarkets posing the main threats to breastfeeding. Given the responsibilities of distributors and industry provided for in the legislation, this work reported abusive marketing practices that negatively impact the human right to adequate food and nutrition of children.

## Resumen

**Objetivo:**

Evaluar las infracciones a la Norma Brasileña de Comercialización de Alimentos para Lactantes y Niños de Primera Infancia, Tetinas, Chupetes y Biberones en el ambiente alimentario minorista alrededor de unidades de salud y centros de educación infantil en Maceió.

**Métodos:**

Se trató de un estudio transversal realizado entre abril de 2022 y marzo de 2023, que efectuó auditorías en todos los puntos de venta de alimentos que comercializaban productos regulados por la Norma en las proximidades de unidades de salud y centros de educación infantil. Los resultados se expresaron en frecuencias.

**Resultados:**

De los 1.176 puntos de venta auditados, el 8,8% presentó infracciones. Las infracciones se concentraron en farmacias (65,0%) y supermercados (26,2%). Al analizar estos dos tipos de establecimientos (n=601), se observó que el 41,6% de los vinculados a redes presentó infracciones. La probabilidad de infracción en redes minoristas fue 6,8 veces mayor (intervalo de confianza del 95% 4,5-10,2) que en establecimientos independientes. Las infracciones más frecuentes ocurrieron en las fórmulas de seguimiento para niños de primera infancia (27,5%), fórmulas infantiles y de seguimiento para lactantes (22,0%) y compuestos lácteos (19,7%). La estrategia promocional más relacionada con las infracciones fue la exposición especial (74,7%).

**Conclusión:**

El ambiente alimentario minorista de Maceió presentó infracciones a la Norma, siendo las redes de farmacias y los supermercados las principales amenazas para la lactancia materna. Dadas las responsabilidades de los distribuidores y la industria previstas en la legislación, este trabajo reporta prácticas comerciales abusivas que impactan negativamente el derecho humano a una alimentación y nutrición adecuadas para los niños.

**Ethical aspects**

This study was conducted in public-access locations and did not involve collecting data from human subjects.:

## Introduction

Since the 1980s, a series of public policies have been implemented in Brazil to protect, promote, and support breastfeeding, including the Brazilian National Breastfeeding Program, the creation of labor laws to protect breastfeeding, and the regulation of human milk banks. Another policy was the Brazilian Code of Marketing of Infant and Toddler Food, Teats, Pacifiers, and Baby Bottles (NBCAL), which regulates the marketing promotion of food and products for infants and children in early childhood (1-3). 

Together, these strategies led to improvements in breastfeeding indicators, particularly between 1986 and 2006 (2). One of these indicators is the prevalence of exclusive breastfeeding for children under 6 months of age, which increased from 2.9% to 37.1% in this period (4). However, between 2006 and 2019, little progress was achieved, especially in the Northeast, where the prevalence of exclusive breastfeeding (39.0%) was below the national prevalence (45.8%) and far from the target of 70.0% established by the World Health Organization and the United Nations Children’s Fund for 2030 (1).

The impact of these policies is compromised by the interference of the infant food industry in public policy governance spaces and by decades of advertising strategies that have opposed the pillars of breastfeeding, altering social norms related to breastfeeding (5-7). The regulation of the marketing of these products sets the basis for constructing breastfeeding-supportive food environments, as it protects those responsible for infants from marketing communications and increases autonomy over choices regarding child nutrition (5,8).

From the perspective of analyzing food environments, the Brazilian Code of Marketing of Infant and Toddler Food, Teats, Pacifiers, and Baby Bottles (NBCAL) is the only law that directly regulates the marketing of food products in retail settings. However, as regulations have become stricter, the industry promotes the marketing of products for infants and children through even more aggressive strategies (8).

Monitoring conducted by public institutions or organized civil society organizations demonstrates that violations of the Code are systematic and reveal the active role of retail in creating food environments unfavorable to breastfeeding, which constitutes a barrier to Brazil meeting its international commitments (5,9).

Despite the growing production (10), studies evaluating the food environment in urban areas remain relatively scarce, with a concentration in the South and Southeast regions. In addition, few are based on primary collection (audits) and address aspects of childhood.

By understanding the factors that interfere with the food environment related to childhood and the violations of legislation, it is possible to produce evidence to support regulatory agencies involved with its monitoring, develop strategies to address the issue, and build a food environment that protects, promotes, and supports breastfeeding. 

The objective of this study was to assess violations of the Brazilian Code of Marketing of Infant and Toddler Food, Teats, Pacifiers, and Baby Bottles (NBCAL) in retail establishments located near health centers and early childhood education centers in Maceió.

## Methods

### Study design and setting

This was a cross-sectional study entitled “Urban Health: Geospatial Analysis on Food and Nutritional Environment in the Spaces Occupied by Children Living in the City of Maceió/Alagoas,” which aimed to characterize the food environment around early childhood education centers and health centers of the city.

The first stage of the research involved georeferencing early childhood education centers and health centers, based on records from the 2021 School Census and the National Health Establishment Registry.

**Figure 1:**
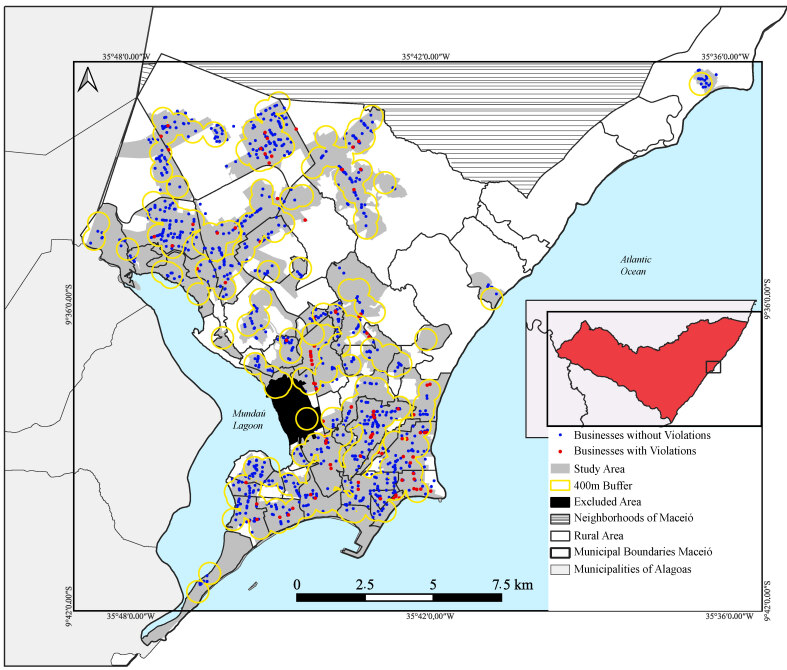
Distribution of establishments according to the occurrence of violations of the Brazilian Code of Marketing of Infant and Toddlers Food, Teats, Pacifiers and Baby Bottles (NBCAL) around early childhood education centers and health centers. Maceió, 2022-2023 (n=1,176)

Next, 400-meter buffers were established around these areas, based on the study by Wilkins and colleagues (11), and the feasibility was validated after a pilot study. To optimize field logistics and align data collection with the city’s administrative units, coverage areas were defined using a network of buffers based on urban census tracts. For this purpose, a census tract was selected when the buffer included 50.0% or more of its area (Figure 1). 

Based on the definition of the coverage areas surrounding early childhood education centers and health centers, protocols were developed to identify and audit food retailers. No rural areas were investigated.

### Data sources: food retail outlets audit 

Data collection took place between April 2022 and March 2023. The data collection instrument was tested with simulated completions and subsequently in a pilot study outside the study coverage area. The team training and the field procedures protocol were conducted based on the Census Taker’s Manual (12).

With the route maps in hand, the teams explored all streets within the coverage areas to identify and audit establishments, employing the ‘gold standard’ method in georeferencing studies (13). The displacement on the streets was carried out by at least two members of the team, one of whom was in charge of guiding the displacement and supporting the others, who were responsible for carrying out the audit.

The audits were conducted based on the “evaluation of commercial establishments” form of the Multi-NBCAL study (14). Audit data, including geographical coordinates, were recorded on smartphones through the KoboToolbox application interface (Kobo, United States). 

During the collection, no interaction was carried out with employees or those responsible for the establishment. The intent of this was to ensure that no changes were made to potential violations after the team was identified.

### Variables and indicators for monitoring violations 

As the objective of this study was to monitor violations of the Code, these analyses included only establishments that marketed a product covered by Law No. 11,265/2006 (3):

- infant formula for infants and follow-up formula for infants
- follow-up infant formulas for early childhood children;
- bottles, nipples, pacifiers, and nipple shields;
- fluid milk, powdered milk, modified milk, and similar plant-based products); and
- complementary foods and cereal-based foods intended for infants or young children, as well as other milk-based or non-milk-based foods or beverages when marketed or otherwise presented as suitable for feeding infants and young children.

Additionally, violations related to dairy compound products were monitored. Although this type of product was developed after the enactment of Law No. 11,265/2006, conceptually, it is also part of the regulated products and can be classified either as “other milk-based foods or beverages” or as part of the milk group (14). To explore violations related to dairy compound products, a specific analysis category was created for these products. 

The monitoring variables were the presence of violations by type of marketing promotion, according to product group. Violations were quantified by establishment and by product, including the identification of manufacturers. Based on this information, three indicators were calculated (Table 1).

**Table 1:** Definition of variables and monitoring indicators of the Brazilian Code of Marketing of Infant and Toddlers Food, Teats, Pacifiers and Baby Bottles (NBCAL) in retail establishments. Maceió, 2022–2023

Monitoring variables	
Violations due to prohibited marketing promotion	Presence of any marketing promotion strategy for infant formula, infant follow-up formula, nipples, pacifiers, bottles, and nipple shields
Violation due to marketing promotion with a non-compliant or missing Ministry of Health’s warning statement	Absence or non-compliance of the warning statement linked to the marketing promotion of follow-up infant formulas for early childhood children, milks and similar products, complementary foods, and dairy compound products “The Ministry of Health warns: this product should not be used for children under 6 (six) months of age, unless expressly indicated by a doctor or nutritionist. Breastfeeding prevents infections and allergies and is recommended until two (2) years of age or older.” “The Ministry of Health informs: After 6 (six) months of age, continue breastfeeding your child and offer new foods.”
Monitoring indicators	
Proportion of food retail outlets with violations	(Number of food retail outlets with violations of the Code/number of food retail outlets regulated by the Code)x100
Proportion of food retail outlets affiliated with retail chains	(Number of food retail outlets affiliated with retail chains/number of food retail outlets regulated by the Code)x100
Proportion of food retail outlets affiliated with retail chains with violations	(Number of food retail outlets affiliated with retail chains with violations of the Code/number of food retail outlets affiliated with retail chains)x100

Marketing promotion is defined as the “a set of informative and persuasive activities carried out by companies responsible for the production, handling, distribution, and marketing, aimed at inducing the purchase or sale of a given product” (3), including dissemination through audiovisual, auditory, and visual media (Decree No. 9,579/2018) (15). The definitions and product groups included in violations for prohibited commercial promotion, as well as those permitted when accompanied by the Ministry of Health’s warning statement, are presented in Table 1.

Marketing promotion strategies were classified into five categories: marketing price promotions, special displays, distribution of informational materials, progressive discounts, and gifts or prize draws. Multiple violations could be registered across more than one product category within a single establishment.

All evaluations were conducted independently by the research team, without the involvement of health surveillance authorities or connections with public administration agencies. Therefore, ‘audit’ refers to the method of primary data collection used in studies of food environments (11), and ‘violations’ to the non-compliance observed by the team concerning the Code.

### Geoprocessing and statistical methods

The violations were analyzed according to the type of establishment, retail network affiliation, product group, marketing promotion strategy, and manufacturer. Establishments were classified according to the Nutrition Environment Measurement Survey for Stores tool (16), which was adapted and validated for use in Brazil (17). Retail network affiliation was considered when the food outlet integrated a set of establishments that belonged to a single company and operated under the same name or brand.

The mapping of the establishments was carried out in QGIS 3.16 (Open Source Geospatial Foundation, United States), using vector files in shapefile (.shp) format extracted from the Brazilian Institute of Geography and Statistics, projected in the SIRGAS2000 reference system. Descriptive statistics were performed using Stata/SE 15 (StataCorp, United States).

## Results

Out of the 50 neighborhoods in Maceió, four had no early childhood education centers or health centers, and two were excluded because they were uninhabited following the socio-environmental disaster caused by Braskem. In total, 44 neighborhoods were within the study coverage area. One did not have establishments with products regulated by the Code.

A total of 1,176 food retail outletswere audited in the vicinity of early childhood education centers (n=270) and health centers (n=83). The most frequent establishments were grocery stores (32.2%), pharmacies (30.3%), and markets/supermarkets (20.7%) (Table 2). In total, 8.8% of the establishments presented some violation of the legislation (Figure 1), with violations concentrated in pharmacies (18.8%) and supermarkets (11.0%) (Table 2).

**Table 2:** Characterization of food retail outlets based on type of business, affiliation with retail chains, and occurrence of violations of the Brazilian Code of Marketing of Infant and Toddlers Food, Teats, Pacifiers, and Baby Bottles (NBCAL). Maceió, 2022–2023 (n=1,176)

Type of business	Regulated food retail outlets n (%)	Food retail outlets with violations n (%)	Food retail outlets affiliated with retail chains n (%)	Food retail outlets affiliated with reta il chains with violations n (%)
Butcher shops, fish markets, and meat-packing plants	32 (2.7)	1 (3.1)	-	-
Candy retail store	9 (0.7)	1 (11.1)	6 (66.7)	1 (16.7)
Market stall or street market stalls	3 (0.2)	-	-	-
Grocery stores	379 (32.2)	3 (0.8)	3 (0.8)	-
Bakeries	114 (9.6)	3 (2.6)	4 (3.5)	-
Greengrocers or produce markets	13 (1.1)	-	1 (7.7)	-
Markets or supermarkets	244 (20.7)	27 (11.0)	47 (19.3)	16 (34.0)
Department stores	17 (1.4)	-	3 (17.6)	-
Pharmacies	357 (30.3)	67 (18.8)	114 (31.9)	51 (44.7)
Children’s product stores	6 (0.5)	0	1 (16.6)	-
Department stores	2 (0.1)	1 (50.0)	2 (100.0)	1 (50.0)
Total	1,176 (100.0)	103 (8.8)	181 (15.3)	69 (38.1)

Focusing on pharmacies and supermarkets (n=601), it was observed that 41.6% of establishments affiliated with retail chains (n=161) had at least one violation (n=67). Among those not affiliated with retail chains, 6.1% presented violations. The probability of identifying a violation in retail chains was 6.8 times higher (95% confidence interval: 4.5; 10.2) compared to establishments not affiliated with a retail chain.

The occurrence of violations was presented by type of violation and marketing strategy, according to product groups covered by the Code (Table 3). Among the 103 establishments that presented any violation, 269 occurrences were identified, with the most frequent records related to the group in which marketing promotion is allowed only with a warning statement, accounting for 65.4% of the violations. The product group with prohibited marketing promotion corresponded to 34.6% of the violations. 

**Table 3:** Frequency of violations of the Brazilian Code of Marketing of Infant and Toddlers Food, Teats, Pacifiers and Baby Bottles (NBCAL) by product group, type of violation, and marketing strategy. Maceió, 2022–2023 (n=1,176)

	Violations by product groups regulated by the Code						
	Infant formula and follow-up formulas for infants n (%)	Teats, pacifiers, bottles and nipple shields n (%)	Infant formula for early childhood children n (%)	Milks and similar products n (%)	Dairy compound products n (%)	Complementary foods n (%)	Total n (%)
Establishments^a^	289 (9.0)	319 (3.5)	210 (21.0)	856 (1.3)	523 (10.3)	669 (2.7)	1,176
**Type of violation^b^**							
Prohibited marketing promotion	59 (22.0)	34 (12.6)	-	-	-	-	269 (100.0)
Marketing promotion with a non-compliant or missing warning statement from the Ministry of Health	-	-	74 (27.5)	27 (10.0)	53 (19.7)	22 (8.2)	
**Marketing promotion strategy^c^**							
Price promotion	20 (15.0)	3 (2.3)	58 (43.6)	7 (5.2)	38 (28.5)	7 (5.3)	133
Special display	40 (19.9)	36 (17.9)	52 (25.9)	13 (6.4)	40 (19.9)	20 (10.0)	201
Distribution of informational material	-	-	3 (75.0)	1 (25.0)	-	-	4
Progressive discount	6 (10.3)	1 (1.7)	30 (51.7)	-	21 (36.2)	-	58
Gifts/ prize draws	1 (16.7)	-	3 (50.0)	2 (33.3)	-	-	6

^a^The n represents the number of establishments that marketed a given product group, and the % indicates the proportion of establishments with violations. ; ^b^Information accounted for considering the occurrence of violations at the establishment level; ^c^Information accounted for considering the occurrence of violations by product. The sum of percentages may exceed 100.0% as a single product could present violations across multiple dimensions of the Code.

The most frequent illegal marketing promotion observed was for follow-up formulas for young children, noted in 21.0% of establishments selling this product, followed by dairy compound products (10.3%) and infant formulas (9.0%) (Table 3).

A total of 402 occurrences of product-related violations connected with marketing promotion strategies were identified (Table 3). The most frequent violation was special display (50.0%), and, in absolute terms, it was the most applied in five of the six product categories. 34 violations were identified per 100 establishments visited. Of the 235 violations in the groups of infant formulas and food products (Table 4), 78.7% occurred in Nestlé products. 

**Table 4:** Frequency of violations of the Brazilian Code of Marketing of Infant and Toddlers Food, Teats, Pacifiers and Baby Bottles (NBCAL) in retail establishments by manufacturer, according to prohibited marketing promotion or promotion with incorrect or missing warning statements, by product group. Maceió, 2022–2023 (n=1,176)

Manufacturers	Product groups regulated by the Code						Total n (%)
	Infant formula and follow-up formula for infants n (%)	Teats, pacifiers, bottles and nipple shields n (%)	Infant formula for early childhood children n (%)	Milks and similar products n (%)	Dairy compound products n (%)	Complementary foods n (%)	
Nestlé	46 (78.0)	-	65 (87.8)	20 (74.1)	34 (64.1)	20 (90.9)	185 (78.7)
Danone	13 (22.0)	-	9 (12.2)	-	19 (35.9)	-	41 (17.4)
Itambé	-	-	-	5 (18.5)	-	-	5 (2.1)
Camponesa	-	-	-	1 (3.7)	-	-	1 (0.4)
Betânia	-	-	-	1 (3.7)	-	-	1 (0.4)
Maizena	-	-	-	-	-	2 (9.1)	2 (0.9)
Total	59 (100.0)	-	74 (100.0)	27 (100.0)	53 (100.0)	22 (100.0)	235 (100.0)
Lolly	-	14 (41.2)	-	-	-	-	14 (41.2)
Lillo	-	7 (20.6)	-	-	-	-	7 (20.6)
Neopan	-	4 (11.8)	-	-	-	-	4 (11.8)
Kuka	-	3 (8.8)	-	-	-	-	3 (8.8)
Mam	-	2 (5.9)	-	-	-	-	2 (5.9)
Philips	-	1 (2.9)	-	-	-	-	1 (2.9)
Fiona	-	1 (2.9)	-	-	-	-	1 (2.9)
Nuk	-	1 (2.9)	-	-	-	-	1 (2.9)
Needs	-	1 (2.9)	-	-	-	-	1 (2.9)
**Total**		34 (100.0)					34 (100.0)

## Discussion

This study demonstrated that pharmacies and supermarkets, especially those linked to retail chains, were the primary sources of Brazilian Code of Marketing of Infant and Toddler Food, Teats, Pacifiers, and Baby Bottles (NBCAL) violations near early childhood education centers and health centers in Maceió. The most frequent violation was the absence or non-compliance of the mandatory warning phrase on products with permitted marketing promotion. The most frequent violations were found in follow-up formulas for young children, followed by infant formulas and milk-based compounds. Special display was the most recorded promotional strategy. Nestlé products had the highest number of violations across all product groups.

Marketing promotion strategies may vary over time, particularly during holidays or the launch of new products. Moreover, pharmacy and supermarket chains coordinate these strategies territorially, when applicable, resulting in systematic and large-scale violations (18). 

As a limitation of this study, although the collection period captured some degree of seasonality, it is not possible to know how changes in the order of audits would have impacted the results. The findings should be interpreted within the context of food environments surrounding early childhood education centers and health centers. Despite covering 42.8% of the urban area, the objective was not to generalize the results to the entire city, where the violation pattern may differ due to the distribution of establishments or neighborhood demographics. 

Strengths include methodological rigor, comprehensiveness, and the census nature of the study, which audits more establishments than any previous research or monitoring of the Brazilian Code of Marketing of Infant and Toddler Food, Teats, Pacifiers, and Baby Bottles (NBCAL), or the International Code of Marketing of Breastmilk Substitutes. This provides robustness to the characterization of violations in contexts with high child circulation 

Breastfeeding is the most cost-effective strategy for child growth and development (7). However, for decades, the food industry has actively sought to undermine this practice through sponsorships, events, residency programs, pediatric departments, harassment of professionals in their workplaces, aggressive advertising, and the marketing model of their products (6,19). 

The volume and multilateral nature of these strategies have altered social norms surrounding breastfeeding since the 1970s, resulting in Brazil’s failure to meet international breastfeeding commitment indicators despite 50 years of public policies (1). Nestlé and Danone, both transnational corporations that employ this business model, reported the highest number of violations (20), with a particular emphasis on the first. 

Nestlé and Danone are transnational oligopolies, and in 2020, they controlled, respectively, 17.1% and 13.5% of the global infant formula market (19). Their growing economic power enables the acquisition and integration of competing companies, as well as substantial investments in advertising and coordinated political-corporate activities, which influence the political and fiscal decisions of governments (8). 

Despite the 20 years of the law, its low priority in the health surveillance agendas of federal agencies may be the result of decades of interference by these corporations. At the national level, in 2006, under the responsibility of the Brazilian Health Regulatory Agency (Anvisa), the Ministry of Health formed a working group to establish national criteria for monitoring the Code (21). However, there are no records in public repositories on referrals from the group, nor on the performance or results of monitoring. In the states and cities, responsible for compliance with the law (20), the low priority of the agenda, the fragility of the health surveillance areas in the face of multiple demands, the insufficient number of professionals and knowledge of the Code are factors that compromise the surveillance capacity.

National monitoring carried out by the International Baby Food Action Network (IBFAN) reveals systematic processes of legislative violations. This study identified patterns similar to the monitoring carried out in 2023 (22), in which the highest frequency of violations by type of product occurred in follow-up infant formulas for early childhood children (26.7%), infant formulas for infants (24.8%) and dairy compound products (12.6%) and, by type of violation, among products for which there marketing promotion (62.1%) is permitted. 

In Salvador, between 2021 and 2022, a similar frequency of violations was identified in infant formulas (up to 6 months of age: 23.7%; 6-12 months of age: 29.5%), follow-up formulas (32.0%) and teats (16.0%), pacifiers (14.6%), bottles (23.4%) and nipple shields (17.2%) (23). The frequency of establishments in Maceió that violated the Code in the marketing promotion of infant formula was lower than observed in Mossoró in 2016 (12.0%) (24) and in the city of Rio de Janeiro in 2017 (16.1%) (18). This study observed fewer violations for complementary foods and nipples, pacifiers, bottles, and nipple shields.

Differences in product grouping made it difficult to compare the other food categories, which, for example, grouped milk, plant-based alternatives, and dairy compound products with follow-up formulas (18), or which used a similar approach but did not include other milk-based or non–milk-based foods in the complementary foods category (24). This study differs from previous studies in terms of the number of violations registered among similar products, including fluid, powdered, modified, and plant-based ones. While the violations varied between 40.0% and 60.0% in these studies, in this research, the frequency was not higher than 10.0%, even after applying different forms of categorization. Considering that data collection occurred at the end of the COVID-19 pandemic, it is plausible that the low frequency of violations is connected to fewer marketing promotions, within a context of increasing food prices and decreased household purchasing capacity (25) Another justification may be associated with the methodological design, as this study audited all establishments marketing any products subject to the legislation, while other studies used convenience sampling. Finally, the possibility that retail establishments in Maceió properly implement the legislation regarding milk and similar products cannot be excluded.

Regarding the types of establishments, pharmacies were identified as the main sources of violations (20,23), especially when affiliated with retail chains (18). 

Retail chains can offer lower prices for marketed products due to their capacity to negotiate large quantities directly with manufacturers, which reduces product costs, and to operate their own logistics networks, which cuts operational expenses. In a capitalist logic and the absence of inspection, retail chains employ illegal marketing practices for products covered by the Code in pursuit of profit maximization. It should be emphasized that this practice does not occur independently of the food industry and other childcare product sectors (19)

A systematic review including 153 studies conducted in nearly 100 countries found that the leading manufacturers of commercial formula are involved in violations of the International Code and that their claims of compliance are misleading (6,8) In Brazil, the industry took advantage of the introduction of a new product — dairy compound products — after the enactment of the Code, in order to circumvent the regulatory legislation (8,28). 

In addition to marketing promotion strategies, the food industry exploits loopholes in legislation to produce labels almost identical to those of infant formulas, to confuse consumers into indirectly associating the two products as if they were similar. Such an approach is called cross-promotion (19). 

Infant formulas are indicated to replace, totally or partially, breast milk, upon diagnosis of specific conditions and prescription of a doctor or nutritionist. Dairy compound products are ultra-processed products, whose composition should present at least 51.0% of dairy ingredients, while 49.0% may include different types of oils, sugars, and food additives (26-27). Therefore, in addition to not resembling infant formulas in any perspective with infant formulas, its composition is expressly contraindicated for children under 2 years of age (27). The most recent scientific evidence does not support the superiority of follow-up infant formulas or dairy compound products over animal milk in terms of infant growth and development (26).

Since it was enacted after Law No. 11,265/2006, the industry has used different arguments to exclude dairy compound products from the scope of the law. However, the product’s visual identity, advertising campaigns, and descriptions of complementary foods place it in this framework (28). To address the distortions caused by the industry, Bill No. 1,407/2023 is currently under consideration in the National Congress, proposing the explicit inclusion of dairy compound products in the Code.

Research of this nature reinforces the importance of health surveillance activities in curbing such practices, especially by predicting fines that can range from BRL 2 thousand to BRL 1.5 million (29). From a conservative perspective, assuming fines for minor violations were applied uniformly across all establishments (disregarding mitigating and aggravating factors), Maceió could have collected approximately BRL 206,000 during the period. As a reference, this amount is 2.6 times higher than the transfers allocated to the city by the Food and Nutrition Fund (FAN), an incentive linked to the National Food and Nutrition Policy (30) 

In conclusion, violations of the regulation were frequent in the food environment surrounding early childhood education centers and health services, especially regarding formulas for young children and dairy compound products, representing a threat to the human right to adequate food and nutrition for children. The occurrence of abusive marketing practices in high-traffic settings used by child caregivers underscores the imperative for health surveillance authorities to rigorously enforce regulations that safeguard breastfeeding and support optimal complementary feeding.
