# Supplementary material for: Marketing violations involving products that compete with breastfeeding in the vicinity of early childhood education centers and health centers: a cross-sectional study based on an audit of the retail food environment, Maceió, 2022–2023
Source: Epidemiol Serv Saude. 2025 Sep 29;34:e20240666. doi: 10.1590/S2237-96222025v34e20240666.en (PMC12479031; doi:10.1590/S2237-96222025v34e20240666.en)
Supplement: Translation

# Translation: Infrações comerciais de produtos concorrentes ao aleitamento materno no entorno de centros de educação infantil e unidades de saúde: estudo transversal baseado em auditoria do ambiente alimentar de varejo, Maceió, 2022-2023

**DOI:** 10.1590/S2237-96222025v34e20240666.pt

**Authors:** Emanuelle Cristina Lins Bastos, Nicole Almeida Conde Vidal, Luan dos Santos Aragão, Gabriel Marx Assunção Costa, Jonas Augusto Cardoso da Silveira

**Affiliations:** 1 Universidade Federal de Alagoas, Programa de Pós-Graduação em Nutrição, Maceió, AL, Brasil.; 2 Universidade Federal do Paraná, Departamento de Nutrição, Curitiba, PR, Brasil

**Keywords:** Espaço Social Alimentar, Aleitamento Materno, Substitutos do Leite Humano, Código Internacional de Comercialização de Alimentos para Lactentes, Estudo Observacional

## Abstract

**Objetivo:**

Avaliar infrações à *Norma Brasileira de Comercialização de Alimentos para Lactentes e Crianças de Primeira Infância, Bicos, Chupetas e Mamadeiras* no ambiente alimentar de varejo ao redor de unidades de saúde e centros de educação infantil de Maceió.

**Métodos:**

Tratou-se de estudo transversal, conduzido entre abril de 2022 e março de 2023, que realizou auditoria em todos os pontos de vendas de alimentos que comercializavam produtos regulados pela Norma no entorno de unidades de saúde e centros de educação infantil. Os resultados foram expressos como frequências.

**Resultados:**

Dos 1.176 pontos de venda de alimentos auditados, 8,8% apresentaram infração. As infrações se concentraram em farmácias (65,0%) e supermercados (26,2%). Ao recortar esses dois tipos de estabelecimentos (n=601), observou-se que 41,6% daqueles vinculados a redes apresentaram infração. A probabilidade de infração em redes de varejo foi 6,8 vezes maior (intervalo de confiança de 95% 4,5-10,2 ) do que em estabelecimentos independentes. As infrações mais frequentes ocorreram nas fórmulas de seguimento para crianças de primeira infância (27,5%), fórmulas infantis e de seguimento para lactentes (22,0%) e compostos lácteos (19,7%). A estratégia promocional mais relacionada às infrações foi a exposição especial (74,7%).

**Conclusão:**

O ambiente alimentar de varejo de Maceió apresentou infrações à Norma, sendo as redes de farmácias e os supermercados as principais ameaças para o aleitamento materno. Dadas as responsabilidades dos distribuidores e da indústria previstas na legislação, este trabalho reportou práticas comerciais abusivas que impactam negativamente o direito humano à alimentação e nutrição adequadas de crianças.

**Aspectos éticos**

Esta pesquisa foi realizada em locais de acesso público e não envolveu a coleta de dados de seres humanos.:

## Introdução

A partir da década de 1980, uma série de políticas públicas foi implementada para proteção, promoção e apoio à amamentação no Brasil, como o Programa Nacional de Incentivo ao Aleitamento Materno, a criação de leis trabalhistas de proteção à amamentação e a regulamentação dos bancos de leite humano. Outra política foi a *Norma Brasileira de Comercialização de Alimentos para Lactentes e Crianças de Primeira Infância, Bicos, Chupetas e Mamadeiras*, que regulamenta a promoção comercial de alimentos e produtos para lactentes e crianças na primeira infância (1-3). 

Em conjunto, essas estratégias resultaram em avanços dos indicadores relativos ao aleitamento materno, especialmente entre 1986 e 2006 (2). Um desses indicadores é a prevalência do aleitamento materno exclusivo em menores de 6 meses de idade que saltou de 2,9% para 37,1% nesse período (4). Entretanto, entre 2006 e 2019, poucos avanços foram conquistados, principalmente no Nordeste, onde a prevalência de aleitamento materno exclusivo (39,0%) esteve abaixo da prevalência nacional (45,8%) e distante da meta de 70,0% estabelecida pela Organização Mundial de Saúde e pelo Fundo das Nações Unidas para a Infância para 2030 (1).

O impacto dessas políticas é comprometido pela interferência da indústria de alimentos para crianças em espaços de governança de políticas públicas e por décadas de estratégias de publicidade antagônicas aos pilares do aleitamento materno, modificando as normas sociais ligadas à amamentação (5-7). A regulação da comercialização desses produtos estabelece bases para a construção de ambientes alimentares protetores do aleitamento materno, pois protege os responsáveis por lactentes da comunicação mercadológica e aumenta a autonomia sobre as escolhas referentes à alimentação infantil (5,8).

Do ponto de vista da análise de ambientes alimentares, a *Norma Brasileira de Comercialização de Alimentos para Lactentes e Crianças de Primeira Infância, Bicos, Chupetas e Mamadeiras* é a única lei que ordena, diretamente, como pode ocorrer a comercialização de um produto alimentício nos espaços de varejo. No entanto, à medida que as regulamentações se tornaram mais rígidas, a indústria promove a comercialização de produtos para lactentes e crianças por meio de estratégias ainda mais agressivas (8).

Monitoramentos realizados por órgãos públicos ou da sociedade civil organizada evidenciam que as infrações à Norma são sistemáticas e demonstram o papel ativo do varejo em construir ambientes alimentares desfavoráveis à amamentação, o que representa uma barreira para que o Brasil cumpra com as metas internacionais (5,9).

Apesar da crescente produção (10), estudos que avaliam o ambiente alimentar nos espaços urbanos ainda são relativamente escassos e se concentram nas regiões Sul e Sudeste. Além disso, poucos se baseiam em coleta primária (auditorias) e abordam aspectos da infância.

Ao compreender os fatores que interferem no ambiente alimentar relacionado à infância e as infrações à legislação, é possível produzir evidências para subsidiar órgãos fiscalizadores envolvidos com seu monitoramento, traçar estratégias para o enfrentamento do problema e construir um ambiente alimentar que proteja, promova e suporte o aleitamento materno. 

Este estudo teve como objetivo avaliar as infrações à *Norma Brasileira de Comercialização de Alimentos para Lactentes e Crianças de Primeira Infância, Bicos, Ch^u^petas e Mamadeiras* nos estabelecimentos comerciais de varejo de Maceió no entorno de unidades de saúde e centros de educação infantil.

## Métodos

### Delineamento e contexto do estudo

Tratou-se de estudo transversal intitulado “Saúde urbana: análise geoespacial sobre ambiente alimentar e nutricional nos espaços ocupados por crianças residentes no município de Maceió/Alagoas”, que teve como objetivo caracterizar o ambiente alimentar no entorno de centros de educação infantil e unidades básicas de saúde do município.

A primeira etapa da pesquisa consistiu no georreferenciamento dos centros de educação infantil e das unidades básicas de saúde, com base nos registros do Censo Escolar 2021 e do Cadastro Nacional de Estabelecimentos de Saúde.

Em seguida, foram traçados raios de 400 metros (buffer) no entorno desses espaços, definido com base no estudo de Wilkins e colaboradores (11), e a viabilidade foi validada após estudo-piloto. Com o objetivo de otimizar a logística de campo e compatibilizar a coleta de dados com unidades administrativas do município, as áreas de abrangência foram definidas a partir de *buffers* em rede com base nos setores censitários urbanos. Para isso, o setor censitário foi selecionado quando o *buffer* incluiu 50,0% ou mais de sua área (Figura 1). 

**Figura 1:** Distribuição de comércios segundo a ocorrência de infrações à *Norma Brasileira de Comercialização de Alimentos para Lactentes e Crianças de Primeira Infância, Bicos, Chupetas e Mamadeiras* no entorno de centros de educação infantil e unidades básicas de saúde. Maceió, 2022-2023 (n=1.176)

A partir da definição das áreas de abrangência nos entornos dos centros de educação infantil e das unidades básicas de saúde, foram estruturados roteiros para a identificação e a auditoria dos comércios de alimentos. Não foram investigadas áreas rurais.

### Fonte dos dados: auditoria nos pontos de venda de alimentos

A coleta de dados ocorreu entre abril de 2022 e março de 2023. O instrumento de coleta foi testado com preenchimentos simulados e, posteriormente, em um estudo-piloto fora da área de cobertura do estudo. O treinamento da equipe e o protocolo de procedimentos em campo foram feitos com base no Manual do Recenseador (12).

Em posse dos mapas com as rotas, as equipes percorreram todas as ruas incluídas nas áreas de abrangência, a fim de identificar e auditar os estabelecimentos, método “padrão-ouro” em estudos de georreferenciamento (13). O deslocamento nas ruas foi realizado por, pelo menos, dois integrantes da equipe, sendo um encarregado de orientar o deslocamento e apoiar aos demais, os quais eram responsáveis pela realização da auditoria.

As auditorias foram conduzidas com base no formulário “avaliação dos estabelecimentos comerciais” do estudo Multi-NBCAL (14). Os dados das auditorias, incluindo as coordenadas geográficas, foram registrados em smartphones por meio da interface do aplicativo KoboToolbox (Kobo, Estados Unidos). 

Durante a coleta, nenhuma interação era realizada com funcionários ou responsáveis pelo estabelecimento. O intuito disso era garantir que não fossem feitas alterações em possíveis infrações após a identificação da equipe.

### Variáveis e indicadores de monitoramento das infrações

Como o objetivo deste estudo foi monitorar as infrações à Norma, estas análises incluíram apenas os estabelecimentos que comercializavam algum produto abrangido pela Lei nº 11.265/2006 (3):

- fórmulas infantis para lactentes e fórmulas de seguimento para lactentes;
- fórmulas infantis de seguimento para crianças de primeira infância;
- mamadeiras, bicos, chupetas e protetores de mamilos;
- leites fluidos, leites em pó, leites modificados e similares de origem vegetal); e
- alimentos de transição e alimentos à base de cereais indicados para lactentes ou crianças de primeira infância, bem como outros alimentos ou bebidas à base de leite ou não, quando comercializados ou de outra forma apresentados como apropriados para a alimentação de lactentes e crianças de primeira infância.

Adicionalmente, foram monitoradas as infrações relativas aos compostos lácteos. Apesar de esse tipo de produto ter sido desenvolvido após a promulgação da Lei nº 11.265/2006, conceitualmente, também faz parte dos produtos regulados, podendo ser classificado como “outros alimentos ou bebidas à base de leite” ou como parte do grupo de leites (14). A fim de explorar infrações sobre os compostos lácteos, criou-se uma categoria específica de análise para esses produtos. 

As variáveis de monitoramento foram a presença de infrações por tipo de promoção comercial, segundo grupo de produtos. As infrações foram quantificadas por estabelecimento e por produto, incluindo a identificação dos fabricantes. A partir dessas informações, foram calculados três indicadores (Tabela 1).

**Tabela 1:** Definição das variáveis e dos indicadores de monitoramento da *Norma Brasileira de Comercialização de Alimentos para Lactentes e Crianças de Primeira Infância, Bicos, Chupetas e Mamadeiras* em estabelecimentos comerciais. Maceió, 2022-2023

Variáveis de monitoramento	
Infração por promoção comercial proibida	Presença de qualquer estratégia de promoção comercial de fórmulas infantis para lactentes, fórmulas infantis de seguimento para lactentes, bicos, chupetas, mamadeiras e protetores de mamilo
Infração por promoção comercial com a frase em desacordo ou sem a frase de advertência do Ministério da Saúde	Ausência ou inadequação da frase de advertência vinculada à promoção comercial de fórmulas infantis de seguimento para crianças de primeira infância, leites e similares, alimentos de transição e compostos lácteos “O Ministério da Saúde adverte: Este produto não deve ser usado para crianças menores de 6 (seis) meses de idade, a não ser por indicação expressa de médico ou nutricionista. O aleitamento materno evita infecções e alergias e é recomendado até os 2 (dois) anos de idade ou mais.” “O Ministério da Saúde informa: após os 6 (seis) meses de idade continue amamentando seu filho e ofereça novos alimentos.”
Indicadores de monitoramento	
Proporção de pontos de venda de alimentos com infrações	(Número de pontos de venda de alimentos com infrações à Norma/número de pontos de venda de alimentos regulados pela Norma)x100
Proporção de pontos de venda de alimentos inseridos em redes de varejo	(Número de pontos de venda de alimentos inseridos em redes de varejo/número de pontos de venda de alimentos regulados pela Norma)x100
Proporção de pontos de venda de alimentos inseridos em redes de varejo com infrações	(Número de pontos de venda de alimentos inseridos em redes de varejo com infrações à Norma/número de pontos de venda de alimentos em redes de varejo)x100

A promoção comercial é definida como o “conjunto de atividades informativas e de persuasão procedente de empresas responsáveis pela produção ou manipulação, distribuição e comercialização com o objetivo de induzir a aquisição ou venda de um determinado produto” (3), incluindo a divulgação, por meios audiovisuais, auditivos e visuais (Decreto nº 9.579/2018) (15). As definições e os grupos de produtos compreendidos nas infrações por promoção comercial proibida e permitida mediante a frase de advertência do Ministério da Saúde foram apresentados na Tabela 1.

As estratégias de promoção comercial foram classificadas em promoção comercial no preço, exposição especial, entrega de material informativo, desconto progressivo e brindes ou sorteio de prêmios. Múltiplas infrações poderiam ser registradas em mais de um grupo de produtos em um estabelecimento.

Todas as avaliações foram realizadas de maneira independente pela equipe de pesquisa, sem participação de autoridades sanitárias ou vínculo com órgãos da Administração Pública. Assim, o termo auditoria se refere ao método de coleta de dados primários utilizado em estudos de ambientes alimentares (11), e o termo infrações, a não conformidade observada pela equipe em relação à Norma.

### Geoprocessamento e métodos estatísticos

As infrações foram analisadas segundo o tipo de estabelecimento, a inserção em rede de varejo, o grupo de produto, a estratégia de promoção comercial e o fabricante. Os estabelecimentos foram classificados de acordo com o instrumento *Nutrition Environment Measurement Survey for Stores* (16), adaptado e validado para o Brasil (17). A inserção em rede de varejo foi considerada quando o ponto de venda de alimentos integrava um conjunto de estabelecimentos que pertenciam a uma única empresa e operavam sob o mesmo nome ou bandeira.

O mapeamento dos estabelecimentos foi realizado no QGIS 3.16 (Open Source Geospatial Foundation, Estados Unidos), utilizando arquivos vetoriais em formato shapefile (.shp) extraídos do Instituto Brasileiro de Geografia e Estatística, projetados no sistema de referência SIRGAS 2000. As estatísticas descritivas foram realizadas no Stata/SE 15 (StataCorp, Estados Unidos).

## Resultados

Dos 50 bairros em Maceió, quatro não apresentavam centros de educação infantil ou unidades básicas de saúde, e dois foram excluídos por estarem desabitados pela catástrofe socioambiental causada pela Braskem. No total, 44 bairros estavam na área de abrangência da pesquisa. Um não apresentou estabelecimentos com produtos regulados pela Norma.

Foram auditados 1.176 pontos de venda de alimentos no entorno dos centros de educação infantil (n=270) e das unidades básicas de saúde (n=83). Os estabelecimentos mais encontrados foram mercearias (32,2%), farmácias (30,3%) e mercados/supermercados (20,7%) (Tabela 2). No total, 8,8% dos comércios apresentaram alguma infração à legislação (Figura 1), estando concentradas em farmácias (18,8%) e supermercados (11,0%) (Tabela 2).

**Tabela 2:** Caracterização dos pontos de vendas de alimentos segundo tipo de comércio, inserção em rede e ocorrência de infrações à *Norma Brasileira de Comercialização de Alimentos para Lactentes e Crianças de Primeira Infância, Bicos, Chupetas e Mamadeiras*. Maceió, 2022-2023 (n=1.176)

Tipo de comércio	Pontos de venda de alimentos regulados n (%)	Pontos de venda de alimentos com infrações n (%)	Pontos de venda de alimentos inseridos em redes de varejo n (%)	Pontos de venda de alimentos inseridos em redes de varejo com infrações n (%)
Açougues, peixarias e frigoríficos	32 (2,7)	1 (3,1)	-	-
Comércio varejista de doces	9 (0,7)	1 (11,1)	6 (66,7)	1 (16,7)
Banca ou barracas de feira	3 (0,2)	-	-	-
Mercearias	379 (32,2)	3 (0,8)	3 (0,8)	-
Padarias	114 (9,6)	3 (2,6)	4 (3,5)	-
Quitandas ou sacolões	13 (1,1)	-	1 (7,7)	-
Mercados ou supermercados	244 (20,7)	27 (11,0)	47 (19,3)	16 (34,0)
Lojas de conveniência	17 (1,4)	-	3 (17,6)	-
Farmácias	357 (30,3)	67 (18,8)	114 (31,9)	51 (44,7)
Lojas de produtos infantis	6 (0,5)	0	1 (16,6)	-
Lojas de departamento	2 (0,1)	1 (50,0)	2 (100,0)	1 (50,0)
Total	1.176 (100,0)	103 (8,8)	181 (15,3)	69 (38,1)

Ao recortar farmácias e supermercados (n=601), observou-se que 41,6% dos estabelecimentos vinculados a redes de varejo (n=161) apresentaram alguma infração (n=67). Entre os que não integravam redes, 6,1% apresentaram infração. A probabilidade de identificar uma infração em redes de varejo foi 6,8 vezes (intervalo de confiança de 95% 4,5; 10,2 ) maior do que em estabelecimentos não inseridos em rede.

Apresentou-se a ocorrência de infrações por tipo de infração e estratégia de promoção comercial, segundo grupos de produtos abrangidos pela Norma (Tabela 3). Nos 103 estabelecimentos que apresentaram alguma violação, foram identificadas 269 ocorrências, sendo os registros mais frequentes aqueles relacionados ao grupo em que é permitida a promoção comercial mediante frase de advertência, totalizando 65,4% das infrações. O grupo de produtos com promoção comercial proibida correspondeu a 34,6% das infrações. 

**Tabela 3:** Frequência de infrações à *Norma Brasileira de Comercialização de Alimentos para Lactentes e Crianças de Primeira Infância, Bicos, Chupetas e Mamadeiras* segundo grupo de produtos, tipo de infração e estratégia de promoção comercial. Maceió, 2022-2023 (n=1.176)

	Infrações por grupos de produtos regulados pela Norma						
	Fórmulas infantis e de seguimento para lactentes n (%)	Bicos, chupetas, mamadeiras e protetores de mamilo n (%)	Fórmulas infantis de seguimento para crianças de primeira infância n (%)	Leites e similares n (%)	Compostos lácteos n (%)	Alimentos de transição n (%)	Total n (%)
Estabelecimentos^a^	289 (9,0)	319 (3,5)	210 (21,0)	856 (1,3)	523 (10,3)	669 (2,7)	1.176
**Tipo de infração^b^**							
Promoção comercial proibida	59 (22,0)	34 (12,6)	-	-	-	-	269 (100,0)
Promoção comercial com a frase em desacordo ou sem a frase de advertência do Ministério da Saúde	-	-	74 (27,5)	27 (10,0)	53 (19,7)	22 (8,2)	
**Estratégia de promoção comercial^c^**							
Promoção comercial no preço	20 (15,0)	3 (2,3)	58 (43,6)	7 (5,2)	38 (28,5)	7 (5,3)	133
Exposição especial	40 (19,9)	36 (17,9)	52 (25,9)	13 (6,4)	40 (19,9)	20 (10,0)	201
Entrega de material informativo	-	-	3 (75,0)	1 (25,0)	-	-	4
Desconto progressivo	6 (10,3)	1 (1,7)	30 (51,7)	-	21 (36,2)	-	58
Brindes/sorteio de prêmios	1 (16,7)	-	3 (50,0)	2 (33,3)	-	-	6

^a^O n representa a quantidade de estabelecimentos que comercializavam um determinado grupo de produtos, e o %, a frequência de estabelecimentos com infrações; ^b^Informação contabilizada considerando a ocorrência de infrações no estabelecimento; ^c^Informação contabilizada considerando a ocorrência de infrações por produto. A soma dos percentuais pode exceder 100,0%, uma vez que um mesmo produto poderia apresentar infrações em diferentes dimensões da Norma.

A promoção comercial ilegal de fórmulas para crianças da primeira infância foi a mais frequente e observada em 21,0% dos estabelecimentos que vendiam esse produto, seguida dos compostos lácteos (10,3%) e fórmulas para lactentes (9,0%) (Tabela 3).

Identificaram-se 402 ocorrências quanto às infrações por produto ligadas às estratégias de promoção comercial (Tabela 3). A infração mais frequente foi a exposição especial (50,0%) e, em termos absolutos, a mais aplicada em cinco das seis categorias de produtos. Foram identificadas 34 violações a cada 100 estabelecimentos visitados. Das 235 infrações nos grupos de fórmulas infantis e produtos alimentícios (Tabela 4), 78,7% ocorreram em produtos da Nestlé. 

**Tabela 4:** Frequência de infrações à *Norma Brasileira de Comercialização de Alimentos para Lactentes e Crianças de Primeira Infância, Bicos, Chupetas e Mamadeiras* nos estabelecimentos por fabricante quanto à promoção comercial proibida ou com frase em desacordo ou sem a frase de advertência, segundo grupo de produtos. Maceió, 2022-2023 (n=1.176)

Fabricantes	Grupos de produtos regulados pela Norma						Total n (%)
	Fórmulas infantis para lactentes e de seguimento para lactentes n (%)	Bicos, chupetas, mamadeiras e protetores de mamilo n (%)	Fórmulas infantis de seguimento para crianças de primeira infância n (%)	Leites e similares n (%)	Compostos lácteos n (%)	Alimentos de transição n (%)	
Nestlé	46 (78,0)	-	65 (87,8)	20 (74,1)	34 (64,1)	20 (90,9)	185 (78,7)
Danone	13 (22,0)	-	9 (12,2)	-	19 (35,9)	-	41 (17,4)
Itambé	-	-	-	5 (18,5)	-	-	5 (2,1)
Camponesa	-	-	-	1 (3,7)	-	-	1 (0,4)
Betânia	-	-	-	1 (3,7)	-	-	1 (0,4)
Maizena	-	-	-	-	-	2 (9,1)	2 (0,9)
Total	59 (100,0)	-	74 (100,0)	27 (100,0)	53 (100,0)	22 (100,0)	235 (100,0)
Lolly	-	14 (41,2)	-	-	-	-	14 (41,2)
Lillo	-	7 (20,6)	-	-	-	-	7 (20,6)
Neopan	-	4 (11,8)	-	-	-	-	4 (11,8)
Kuka	-	3 (8,8)	-	-	-	-	3 (8,8)
Mam	-	2 (5,9)	-	-	-	-	2 (5,9)
Philips	-	1 (2,9)	-	-	-	-	1 (2,9)
Fiona	-	1 (2,9)	-	-	-	-	1 (2,9)
Nuk	-	1 (2,9)	-	-	-	-	1 (2,9)
Needs	-	1 (2,9)	-	-	-	-	1 (2,9)
**Total**		34 (100,0)					34 (100,0)

## Discussão

Este estudo demonstrou que as principais fontes de violação à Norma no entorno de centros de educação infantil e unidades básicas de saúde em Maceió foram as farmácias e os supermercados, especialmente quando vinculados a redes de varejo. A infração mais frequente foi a ausência ou inadequação da frase de advertência obrigatória em produtos com promoção comercial permitida. Os produtos que apresentaram maiores registros de infrações foram as fórmulas infantis de seguimento para crianças de primeira infância, seguidas pelas fórmulas infantis e de seguimento para lactentes e compostos lácteos. A estratégia de promoção comercial mais registrada foi a exposição especial. Produtos Nestlé apresentaram o maior número de infrações, com ocorrência em todos os grupos alimentícios.

Estratégias de promoção comercial podem ter alta variabilidade no tempo, especialmente em datas comemorativas ou no lançamento de novos produtos. Além disso, redes de farmácias e supermercados operam tais estratégias de maneira coordenada em seus territórios, quando é o caso, cometendo infrações de maneira sistemática e em larga escala (18). 

Como limitação deste estudo, apesar de o período de coleta ter permitido incorporar algum grau de sazonalidade, não é possível saber como mudanças na ordem das auditorias teriam impactado os resultados. É fundamental interpretar esses resultados considerando seu escopo sobre o ambiente alimentar nos entornos dos centros de educação infantil e unidades básicas de saúde. Apesar da cobertura de 42,8% da área urbana, não foi o objetivo generalizar os resultados para todo o município, onde o padrão de infrações pode ser diferente pela distribuição dos estabelecimentos ou pela demografia dos bairros. 

Como ponto forte, destacam-se o rigor metodológico, a abrangência e o caráter censitário do estudo, auditando mais estabelecimentos que qualquer pesquisa ou monitoramento prévio da *Norma Brasileira de Comercialização de Alimentos para Lactentes e Crianças de Primeira Infância, Bicos, Chupetas e Mamadeiras* ou do *Código Internacional de Comercialização de Substitutos do Leite Materno*. Isso confere robustez sobre a caracterização das infrações nesses contextos de alta circulação de crianças. 

O aleitamento materno é a estratégia mais custo-efetiva para o crescimento e o desenvolvimento infantil (7). Entretanto, há décadas a indústria de alimentos busca ativamente minar essa prática por meio de patrocínios, eventos, programas de residência, departamentos de pediatria, assédio de profissionais em seus locais de trabalho, peças publicitárias agressivas e do modelo de comercialização de seus produtos (6,19). 

O volume e a multilateralidade dessas estratégias modificaram as normas sociais ligadas à amamentação desde os anos 1970, de tal modo que meio século de políticas públicas não foram suficientes para que o Brasil apresentasse indicadores de aleitamento materno dentro das metas internacionais (1). Nestlé e Danone, ambos conglomerados transnacionais que praticam esse modelo de negócio, apresentaram o maior número de infrações (20) – com destaque para a primeira. 

Nestlé e Danone são transnacionais oligopolistas e, em 2020, dominavam, respectivamente, 17,1% e 13,5% do mercado global de fórmulas infantis (19). O crescente poder econômico permite a aquisição e incorporação de empresas concorrentes e investimentos vultuosos em publicidade e atividades político-corporativas coordenadas para exercer influência sobre decisões políticas e fiscais de governos (8). 

Apesar dos 20 anos da lei, a baixa prioridade desta nas agendas das vigilâncias sanitárias dos entes federativos pode ser resultado das décadas de interferências dessas corporações. Em nível nacional, em 2006, sob responsabilidade da Agência Nacional de Vigilância Sanitária, o Ministério da Saúde formou um grupo de trabalho para estabelecer critérios para o monitoramento nacional da Norma (21), porém não há registros em repositórios públicos sobre os encaminhamentos desde grupo, tampouco da realização ou dos resultados do monitoramento. Nos estados e municípios – responsáveis pelo cumprimento da lei (20) –, a baixa prioridade da agenda, a fragilidade das áreas de vigilância sanitária diante de múltiplas demandas, o número insuficiente de profissionais e o conhecimento insuficiente da Norma são fatores que comprometem a capacidade de fiscalização.

Monitoramentos nacionais realizados pela Rede Internacional em Defesa do Direito de Amamentar descrevem processos sistemáticos de infrações à legislação. Este estudo identificou padrões similares ao monitoramento realizado em 2023 (22), no qual as maiores frequências de infrações por tipo de produto ocorreram nas fórmulas infantis de seguimento para crianças de primeira infância (26,7%), fórmulas infantis para lactentes (24,8%) e compostos lácteos (12,6%) e, por tipo de infração, entre os produtos em que pode haver promoção comercial (62,1%). 

Em Salvador, entre 2021 e 2022, identificou-se frequência similar de infrações em fórmulas infantis (até 6º mês: 23,7%; 6º-12º mês: 29,5%), fórmulas de seguimento (32,0%) e de bicos (16,0%), chupetas (14,6%), mamadeiras (23,4%) e protetores de mamilo (17,2%) (23). A frequência de estabelecimentos em Maceió que violaram a Norma na promoção comercial de fórmulas infantis foi menor do que observado em Mossoró em 2016 (12,0%) (24) e no município do Rio de Janeiro em 2017 (16,1%) (18). Neste estudo, foi observada a menor frequência de estabelecimentos com alguma infração para os alimentos de transição e dos bicos, chupetas, mamadeiras e protetores de mamilo.

As formas de agregação dos produtos dificultaram a comparação dos outros grupos de alimentos, que, por exemplo, agruparam os leites, similares de origem vegetal e compostos lácteos às fórmulas de seguimento (18), ou que utilizaram abordagem similar, porém deixaram os outros alimentos à base de leite ou não agrupados com os alimentos de transição (24). Este estudo difere desses trabalhos em relação ao número de infrações registradas no grupo dos leites fluidos, em pó, modificados e similares de origem vegetal. Ao passo que nesses estudos as violações variaram entre 40,0% e 60,0%, nesta pesquisa a frequência não foi superior a 10,0%, mesmo após a aplicação das diferentes formas de categorização. Ao considerar que a coleta de dados ocorreu no final da pandemia de covid-19, conjectura-se que a baixa frequência de infrações possa estar ligada à menor frequência de promoções comerciais, dados o contexto de aumento do preço dos alimentos e a queda nas aquisições diante da redução dos orçamentos familiares (25). Outra justificativa pode estar relacionada ao desenho metodológico, uma vez que este estudo auditou todos os estabelecimentos que comercializavam qualquer produto abrangido pela legislação, enquanto os demais utilizaram amostras por conveniência. Por fim, não se pode descartar a perspectiva de que os estabelecimentos de Maceió implementem adequadamente a legislação para leites e similares.

Quanto aos tipos de estabelecimentos, as farmácias foram identificadas como as principais fontes de infrações (20,23), especialmente quando vinculadas a redes de varejo (18). 

Redes são capazes de oferecer preços mais baixos pelos produtos comercializados devido à sua capacidade de negociar grandes quantidades diretamente com a indústria, reduzindo o valor dos produtos, e possuir redes próprias de logística, reduzindo os custos operacionais. Na lógica capitalista, e diante da ausência da fiscalização, as franquias se utilizam de práticas ilegais de comercialização de produtos abrangidos pela Norma em busca da maximização dos lucros. Ressalta-se que essa prática não acontece de forma isolada da indústria de alimentos e demais produtos de puericultura (19).

Uma revisão sistemática com 153 estudos que avaliaram as violações ao Código Internacional em quase 100 países mostrou que os principais fabricantes de fórmulas comerciais estão envolvidos nas infrações e que suas alegações de conformidade são enganosas (6,8). No Brasil, a indústria se utilizou da introdução de novo produto no mercado após a promulgação da Norma – os compostos lácteos –, a fim de burlar a legislação regulatória (8,28). 

Além das estratégias de promoção comercial, a indústria de alimentos aproveita-se de brechas na legislação para produzir rótulos quase idênticos aos das fórmulas infantis, com objetivo de confundir o consumidor a associar indiretamente os dois produtos como se fossem similares. Tal abordagem é chamada promoção cruzada (19). 

Fórmulas infantis são indicadas para substituir, total ou parcialmente, o leite materno, mediante diagnóstico de condições específicas e prescrição de médico ou nutricionista. Os compostos lácteos são produtos ultraprocessados, cuja composição deve apresentar, no mínimo, 51,0% de ingredientes lácteos, enquanto os 49,0% podem incluir diferentes tipos de óleos, açúcares e aditivos alimentares (26-27). Portanto, além de não se assemelhar em qualquer perspectiva com fórmulas infantis, sua composição é expressamente contraindicada para menores de 2 anos de idade (27). As evidências científicas mais recentes não dão suporte à superioridade de fórmulas infantis de seguimento ou compostos lácteos, em relação ao leite animal, para o crescimento e o desenvolvimento infantil (26).

Por ser posterior à Lei nº 11.265/2006, a indústria utiliza diferentes argumentos para posicionar os compostos lácteos fora da sua abrangência. No entanto, a identidade visual do produto e das campanhas publicitárias e a descrição dos alimentos de transição os colocam nesse arcabouço (28). A fim de corrigir as distorções geradas pela indústria, tramita, no Congresso Nacional, o Projeto de Lei nº 1.407/2023, que propõe a explicitação dos compostos lácteos na Norma.

Pesquisas dessa natureza reforçam a importância das atividades da vigilância sanitária para coibir tais práticas, especialmente pela previsão de multas que podem variar de R$ 2 mil a R$ 1,5 milhão (29). Em uma perspectiva conservadora, na qual se aplica multas referentes a infrações leves em todos os estabelecimentos (desconsiderando atenuantes e agravantes), Maceió poderia ter arrecadado no período R$ 206 mil. Como referência, esse valor é 2,6 vezes maior que os repasses previstos para o município pelo Financiamento das Ações de Alimentação e Nutrição, incentivo ligado à Política Nacional de Alimentação e Nutrição (30). 

Em conclusão, foram frequentes as violações à norma no ambiente alimentar em torno de centros de educação infantil e serviços de saúde, especialmente das fórmulas para crianças de primeira infância e dos compostos lácteos, representando ameaça ao direito humano à alimentação e à nutrição adequadas de crianças. A presença de práticas comerciais abusivas em espaços de alta circulação de pessoas responsáveis pelo cuidado de crianças é um chamado para que as vigilâncias sanitárias cumpram seu papel na proteção ao aleitamento materno e na alimentação complementar saudável.
